# Claimed Effects, Outcome Variables and Methods of Measurement for Health Claims Proposed Under European Community Regulation 1924/2006 in the Framework of Maintenance of Skin Function

**DOI:** 10.3390/nu10010007

**Published:** 2017-12-22

**Authors:** Daniela Martini, Donato Angelino, Chiara Cortelazzi, Ivana Zavaroni, Giorgio Bedogni, Marilena Musci, Carlo Pruneti, Giovanni Passeri, Marco Ventura, Daniela Galli, Prisco Mirandola, Marco Vitale, Alessandra Dei Cas, Riccardo C. Bonadonna, Sergio Di Nuzzo, Maria Beatrice De Felici, Daniele Del Rio

**Affiliations:** 1 The Laboratory of Phytochemicals in Physiology, Department of Food and Drug, University of Parma, 43125 Parma, Italy; daniela.martini@unipr.it (D.M.); donato.angelino@unipr.it (D.A.); 2 Department of Medicine and Surgery, Section of Dermatology, University of Parma, 43125 Parma, Italy; chiara.cortelazzi@gmail.com (C.C.); sergio.dinuzzo@unipr.it (S.D.N.); bea.defelici@gmail.com (M.B.D.F.); 3 Department of Medicine and Surgery, Division of Endocrinology, University of Parma, 43125 Parma, Italy; ivana.zavaroni@unipr.it (I.Z.); alessandra.deicas@unipr.it (A.D.C.); riccardo.bonadonna@unipr.it (R.C.B.); 4 The Azienda Ospedaliera Universitaria of Parma, Division of Endocrinology, 43125 Parma, Italy; 5 Clinical Epidemiology Unit, Liver Research Center, Basovizza, 34149 Trieste, Italy; giorgiobedogni@gmail.com; 6 Department of Food and Drug, University of Parma, 43125 Parma, Italy; marilena.musci@unipr.it; 7 Department of Medicine and Surgery, Clinical Psychology Unit, University of Parma, 43125 Parma, Italy; carlo.pruneti@unipr.it; 8 Department of Medicine and Surgery, University of Parma, Building Clinica Medica Generale, 43125 Parma, Italy; giovanni.passeri@unipr.it; 9 Laboratory of Probiogenomics, Department of Chemistry, Life Sciences and Environmental Sustainability, University of Parma, 43125 Parma, Italy; marco.ventura@unipr.it; 10 Department of Medicine and Surgery, Sport and Exercise Medicine Centre (SEM), University of Parma, 43125 Parma, Italy; daniela.galli@unipr.it (D.G.); prisco.mirandola@unipr.it (P.M.); marco.vitale@unipr.it (M.V.)

**Keywords:** health claim, outcome variable, method of measurement, skin health

## Abstract

Evidence suggests a protective role for several nutrients and foods in the maintenance of skin function. Nevertheless, all the requests for authorization to use health claims under Article 13(5) in the framework of maintenance of skin function presented to the European Food Safety Authority (EFSA) have received a negative opinion. Reasons for such failures are mainly due to an insufficient substantiation of the claimed effects, including the choice of inappropriate outcome variables (OVs) and methods of measurement (MMs). The present paper reports the results of an investigation aimed at collecting, collating and critically analyzing the information with relation to claimed effects (CEs), OVs and MMs related to skin health compliance with Regulation 1924/2006. CEs, OVs and MMs were collected from both the EFSA Guidance document and from the authorization requests of health claims under Article 13(5). The critical analysis of OVs and MMs was based on a literature review, and was aimed at defining their appropriateness (alone or in combination with others) in the context of a specific CE. The results highlight the importance of an adequate choice of OVs and MMs for an effective substantiation of the claims.

## 1. Introduction

Skin represents the most external layer of the organism, and forms an effective barrier between the body and the environment [1]. Skin functions are extremely important and fall in several different categories: resistance to chemical and physical insults, defense from parasites and general poisons, regulation of body water, body temperature, oxygen absorption, and excretion of potentially toxic compounds (i.e., urea) [2,3]. All these functions are possible because of the particular structure of the skin, which consists of at least three main layers: (i) The epidermis, the most external layer, in direct contact with the environment, is formed by a *stratum germinativum* that constantly produces melanin and renews keratinocytes, and a *stratum corneum* consisting of dead cells, where keratin is stored, and which protects the layers underneath; (ii) The dermis, formed by epithelial tissue containing hair follicles, lymph and blood vessels; (iii) The subcutis, where blood vessels, nerves, glands and muscle fibers reside [1]. The cells in the structure are strictly connected by junctions (tight, gap, desmosomes, etc.), which allow the crosslinking of layers, thereby creating a strict and resistant mesh with selective permeability to nutrients and other compounds.

A wide range of diseases, including psoriasis, dermatitis, burns, ulcers, autoimmune disorders and cancer, can originate from alterations of the skin barrier induced by physical or chemical insults, by microorganisms, and by inflammatory processes that activate the immune system [4].

It has been estimated that at least 20% of the world population is affected by some sort of skin disorder requiring medical attention, i.e., viral or bacterial eczema, acne and infections [5]. Hay et al. highlighted that skin disorders are the fourth leading cause of non-fatal burden diseases in terms of years lost in disability [6], and statistics evidenced an 8.5% increase of death from skin disorders, and a 58.4% increase from malignant skin melanoma during the period 1990–2010 [7].

Lifestyle has been recognized as a relevant factor influencing the onset and the development of skin disorders. A major focus has been given to the relationship of body weight and lipid-related skin disease development. Randomized clinical trials (RCTs) have shown that physical activity and diet are strictly related to a decrease of psoriasis severity [8], as well as an improvement in the “dermatology life quality index” [9]. Dietary patterns, i.e., low glycemic load diets, enhanced the reduction of follicular sebum overflow and skin surface triglycerides, and increased the ratio of saturated/monounsaturated fatty acids [10]. However, as authors have concluded that it was not possible to attribute these effects to diet composition or weight loss, the role of the diet on sebum composition needs to be further investigated.

Some investigations have focused on the role of individual components of food, such as bioactive compounds, in skin health RCTs that have considered a lycopene-enriched tomato paste [11], a high flavanol-3-ols drink [12], or a green tea polyphenol beverage [13], showing significant reductions of erythema formation in healthy volunteers. Nevertheless, evidence of a direct effect of both single components and the whole diet in affecting skin-related outcomes is still extremely weak.

The current scientific evidence has convinced stakeholders to submit requests for authorization of health claims to the European Food Safety Authority (EFSA). Some health claims pertinent to Article 13(1) of the Regulation 1924/2006 have been approved (ec.europa.eu/nuhclaims/), whereas all the requests related to Article 13(5) regarding skin function have received a negative opinion. The main reasons for the negative opinions concern the insufficient characterization of the food item and its constituents, the choice of an inappropriate (e.g., vague) claimed effect and/or target population, and most of all the inadequate substantiation of the claim through well-designed and well-performed RCTs [14,15]. Many parameters may affect the quality of an RCT, such as the use of a placebo-controlled approach, the calculation of the sample size, the statistical analysis, and the choice of outcome variables (OVs) and/or their methods of measurements (MMs). For these reasons, the present work aims to collect, collate and critically analyze the information in relation to claimed effects (CEs), OVs and MMs, in the context of maintenance of normal skin function.

## 2. Search Strategy

This manuscript refers to the critical analysis of OVs and MMs collected from the EFSA Guidance on the scientific requirements for health claims related to bone, joints, skin, and oral health (EFSA 2011), from the requests for authorization of health claims under Articles 13(5) and 14 of Regulation 1924/2006 related to skin health (ec.europa.eu/nuhclaims/), and from comments received during public consultations. Adopting the decision tree described in Martini et al. (2016) [14], 3 claimed effects with 21 OVs were evaluated under Article 13(5). No disease risk reduction claims and no health claims referring to children’s development had been proposed under the Article 14. For each OV, a database of references was created on PubMed based on the keywords defined by each OV, allowing a specific critical analysis of the OVs and the MMs (Table 1). The literature databases were reviewed and used for the critical evaluation of each OV and MM. The critical evaluation of OVs and MMs was performed taking into account their relevance in the framework of randomized controlled trials. For MMs, the two main parameters that were considered were (i) being a gold standard method; and (ii) the field acceptance. Moreover, we considered the following parameters in agreement with Fitzpatrick et al. [16].

- Appropriateness
- Reliability
- Validity
- Responsiveness
- Precision
- Interpretability
- Acceptability
- Feasibility

**Figure 1:**
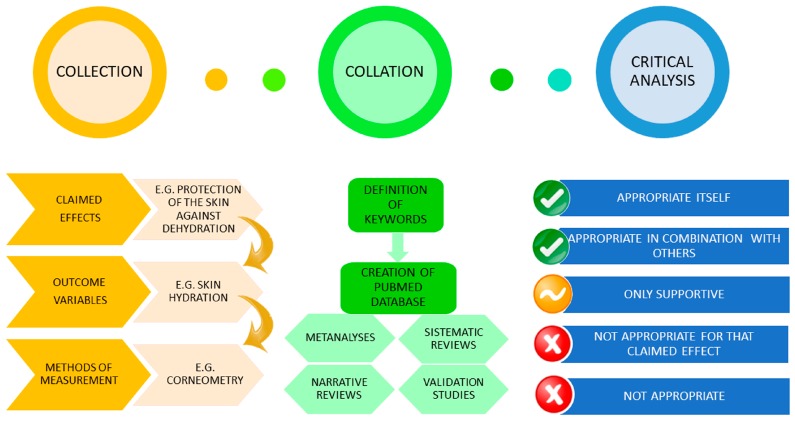
Collection, collation and critical evaluation of information in relation to claimed effects, outcome variables and methods of measurement in the framework of maintenance of skin function: flow chart of the project.

Each OV and related MM was ranked in one of the following categories: (i) appropriate alone; (ii) appropriate only in combination with other OVs or MMs; (iii) not appropriate per se; (iv) not appropriate in relation to the specific claimed effect proposed by the applicant(s); (v) not appropriate alone, but useful as supportive evidence for the scientific substantiation of the claimed effect. The index at the beginning of this paper lists the OVs and the respective MMs from the most to the least appropriate, if a ranking was applicable. The flow chart of the project is shown in Figure 1.

## 3. Critical Evaluation for Function Claims 13(5)

### 3.1. Protection of the Skin Against Dehydration

Skin is the largest organ of our body, and represents a physical barrier capable of holding water, as well as limiting the penetration of chemical substances, microorganisms and radiation from the environment. The ability of the skin to prevent the loss of body fluids is mainly attributed to the role of the *stratum corneum* (SC), an outer layer of the epidermis, formed by several deposits of dead keratinocytes, which acts as a physical barrier, limiting the loss of water and avoiding skin dehydration [1,17]. In particular, the presence of keratine and ceramides allows the formation of a compact layer that prevents the entrance of external particles and at the same time retains the body fluids under the epidermal layer [17].

Nevertheless, the use of aggressive products, such as detergents and surfactants, or exposure to environmental agents (e.g., sun, wind, cold) may compromise the structure and the function of SC, leading to a visible onset of the appearance of dehydration, e.g., dry and rough skin, desquamation. For these reasons, a compromised skin barrier may enhance the penetration of a wide range of substances/pathogens leading to pathological processes. Consequently, the maintenance of its permeability is essential and highly beneficial.

#### 3.1.1. Transepidermal Water Loss

Transepidermal water loss (TEWL) has been recognized for over half a century as an in vivo parameter for assessing the skin barrier function. TEWL can be defined as the outward permeation of condensed water across the SC via diffusion, excluding other forms of water loss, such as perspiration [17]. It is expressed as grams of water per unit area of skin per unit of time (g/m^2^/h).

To evaluate the appropriateness of TEWL as an OV for the protection of skin against dehydration, database 1 was generated (see Table 1).

TEWL is one of the most important parameters for evaluating the permeability function of the skin. A low TEWL is generally a characteristic feature of an intact skin function and a high hydration of the SC. Conversely, an elevated TEWL value is typically correlated with low hydration of the SC, and with an impaired epidermal barrier. This can be associated to several skin diseases, such as atopic dermatitis, or to contact with aggressive substances, such as solvents and detergents [18].

Based on these considerations, TEWL is an appropriate outcome measure to use for the substantiation of health claims in the context of protection of the skin against dehydration.

##### Tewameter^®^

Being the flux of condensed water diffusing through the skin, there are no direct methods for measuring TEWL. Nevertheless, there are two different indirect methods of measurement: open-chamber and closed-chamber methods [19]. Open chambers are open to the surrounding atmosphere. The closed-chamber method is a more recent methodology/procedure, developed to avoid the effect of external air convection and turbulence but, compared to the open chambers, have the disadvantage of requiring a purge after each measurement because of the accumulation of humidity and water vapor.

TEWL assessment can be performed using different instruments and, among them, the Tewameter^®^ (Courage-Khazaka Electronic, Cologne, Germany), an open-chamber instrument, is one of the most widely accepted and employed methods for the measurement of TEWL. Therefore, most of the scientific literature on TEWL refers to this apparatus. 

Open-chamber methods are preferable for the evaluation of skin barrier function, but must occur in a room with standardized conditions (i.e., with standardized temperature and relative humidity) [19].

In these open-chamber methods, TEWL is calculated by measuring the water vapor pressure (VP) gradient immediately above the surface of the skin, calculated as the difference in VP between two distinct points aligned perpendicularly to the skin surface.

The measuring principle of the instrument is that the vapor pressure gradient above the skin surface is proportional to the difference between the vapor pressures measured at two different heights located perpendicularly above the skin surface. With this aim, a probe, consisting of an open cylinder, is placed on the skin. The probe indirectly measures the density gradient of the water evaporation from the skin by two pairs of sensors inside the hollow cylinder. A microprocessor analyzes the values, and expresses the evaporation rate in g/h/m^2^.

TEWL is one of the most measured parameters used in cosmetology to assess the efficacy of moisturizing cosmetics. It can be evaluated in combination with the use of irritants like sodium lauryl sulphate, sodium hydroxide or dimethylsulfoxide, to identify irritant reactions.

It is also worth noting that several and various conditions can influence the value of TEWL, such as ambient temperature, relative humidity, topical products, skin damage or diseases, probe position, sweating, smoking, age, sex, and skin sites [17,18]. Nevertheless, many of these factors can be controlled or minimized by using a well-developed study design. The Tewameter^®^ allows an accurate and quick measurement of the TEWL. Limitations of the Tewameter^®^ include the slight overestimation of the resulting values in comparison to other methods [17], and the variations of results among different instruments of the same type. Despite these limitations, the Tewameter^®^ is one of the most used instruments in RCT.

In conclusion, the Tewameter^®^ is an appropriate method to use for the measurement of TEWL.

#### 3.1.2. Skin Hydration

Skin hydration is defined as the water content of the epidermis and the dermis. An adequate skin hydration is considered a very important factor in skin health. The epithelium remains flexible when it contains 10–20% water, and several substances may contribute to maintaining a balance in the skin homeostasis, such as natural moisturizing factor and some intercellular lipids of the SC [20,21]. All these factors synergize to preserve the adequate skin hydration and the barrier function of the epidermis as well as to prevent TEWL.

Superficial lipids create a filter for interaction with the external environment, and have been found to serve as water modulators in the SC.

The correct amount of water in the SC plays an important role on softness, smoothness and elasticity of the skin and allows maintenance of the typical barrier function of the skin, thus avoiding penetration of substances and microbes. Conversely, dry skin is generally linked to a reduced water content of the SC, and is associated with a rough surface, modifications of the lipid content or profile and of the permeability of the skin [21].

To evaluate the appropriateness of skin hydration as an OV for the protection of skin against dehydration, database 2 was generated (see Table 1).

An appropriate amount of water in the skin is important for the maintenance of its normal structure and properties, including adequate hydration and elasticity of the skin [22]. Moreover, skin hydration has been used extensively as an index of skin barrier function. An altered skin barrier function may modify the permeability function of the skin and facilitate the loss of body fluids and the penetration of chemicals and allergens.

Several diseases are associated to an impaired epidermal barrier, including atopic dermatitis, psoriasis, eczema, ichthyosis, and sensitive skin/rosacea [21]. Skin hydration can also decrease after the contact with aggressive cosmetics or professional substances leading to desiccation of SC with alteration of the normal epidermal barrier.

In conclusion, skin hydration is an appropriate outcome variable to use for the substantiation of health claims in the context of protection of the skin against dehydration. However, the simultaneous measure of TEWL is preferable, and provides a more complete and accurate measure of the hydration state of the skin.

##### Corneometry

Parameters related to skin hydration may be measured by considering the electrical properties that are dependent on the water content of the SC. Many commercially available instruments, based on principles of measuring skin capacitance, conductance, or impedance, have been widely used for this purpose [18,23].

Corneometry is one of the most commonly used techniques for measuring skin hydration, as well as water-holding capacity, as a surrogate measure of skin hydration.

The Corneometer^®^ (Courage-Khazaka Electronic, Cologne, Germany) measures skin hydration by determining the skin capacitance with the use of probes [23]. The measurement is based on the difference between the dielectric constant of water and other substances by measuring the capacitance of a dielectric medium. The Corneometer^®^ measures the change in the dielectric constant due to skin surface hydration by modulating the capacitance of a precision capacitor. The depth of measurement is quite low, as it reaches the first 10–20 μm of the SC.

One of the techniques that can be used is the water sorption-desorption test, which consists of the hydration of the skin with water followed by the observation of the subsequent dehydration activity by means of serial recording with electrical instruments.

The instrument delivers values expressed in arbitrary units (AU) varying from 0 to 120, where a higher value indicates a more hydrated skin with values >40 indicating adequate skin hydration [24].

Similar to other electrical measurements, corneometry has become very popular because of the relative low cost and ease of use; in fact, corneometry measurements are very rapid, and the modern probes enable temperature stability. In addition, the technique minimizes the subjectivity typical of visual scoring methods, and therefore allows greater accuracy and reproducibility [25,26].

Moreover, although several experimental and instrumentation-related factors also influence measurements (e.g., skin temperature, sweating, ambient temperature and humidity), many of these factors can be controlled or minimized by using a well-developed study design and specific standardized-condition rooms.

In conclusion, corneometry is an appropriate non-invasive method that can be used for the measurement of skin hydration and water-holding capacity, especially if in association with other skin parameter measurements, e.g., TEWL. In fact, as these are all indirect parameters, their association can lead to more meaningful results.

#### 3.1.3. Skin Dryness

Skin dryness is a common condition in which skin loses its homeostasis, and there is an impaired epidermal barrier. Many causes can contribute to this condition, such as exposure to aggressive solvents, frequent hand washing, specific occupational activities, or skin diseases (allergic or atopic dermatitis). Aging and hormonal changes can determine skin dryness, too.

Skin dryness is often called “xerosis”, a term used in dermatology to indicate any condition of abnormal dry skin [27].

Pruritus is the most important symptom of skin dryness.

To evaluate the appropriateness of evaluation of skin dryness by an expert evaluator as an OV for the protection of skin against dehydration, database 2 was generated (see Table 1).

Daily insults from the environment can lower the water content of the *stratum corneum*, which impairs the enzymatic function required for normal desquamation, leading to a dry and flaky skin. Therefore, skin dryness can be considered to be an indirect measure of skin hydration.

However, dry skin is associated with major signs such as scaling, roughness, cracks and redness, and thus the evaluation of skin dryness cannot disregard the evaluation of these signs to obtain the overall dry skin score (ODS), a scoring scale combining all these major and minor signs of dry skin [28]. 

In conclusion, the skin dryness score is an appropriate outcome measure to be used for the substantiation of health claims in the context of protection of the skin against dehydration.

However, the combination with additional outcome measures such as TEWL is essential to obtain a more reliable and objective result. 

##### Evaluation of Skin Dryness by an Expert Evaluator

The evaluation of skin dryness can be self-assessed by the subject or performed by an expert evaluator. In the first case, the evaluation can be influenced by subjective feelings, such as itching, while the evaluation by experts is based on objective criteria. 

Usually, as recommended in the guidance published by The European Group on Efficacy Measurement of Cosmetics and other Topical Products, the expert evaluator can use a scoring scale combining all the effects of dry skin and judging it from 0 (absent) to 4 (advanced roughness, redness and cracks) [28]. If necessary, a specified symptom sum score can be used, using an evaluation of the main signs of xerosis (scaling, roughness, redness and cracks) in selected anatomical regions. Finally, an evaluation of the whole skin, as well as specifically at different sites (e.g., head/neck, upper and lower extremities, trunk), can be performed.

Moreover, dryness is a condition often associated with several dermatological diseases, in particular atopic dermatitis (also known as atopic eczema). Different scores have been developed to evaluate and classify disease grade: Eczema Area and Severity Index, Scoring Atopic Dermatitis, Objective Component of Score, Investigator Global Assessment, Atopic Dermatitis Severity Index and Body Surface Area [29]. Simultaneous use of two or more scores is a reliable assessment of disease severity.

The evaluation of dry skin by an expert evaluator is preferable compared to self-evaluation, because it is not influenced by subjective feelings such as itching and thus is more reproducible. Nevertheless, most of these scores consider patient’s symptoms (as pruritus) and the effect of the disease on quality of life.

In conclusion, the evaluation of dry skin by an expert evaluator is an appropriate method to use for the measurement of skin dryness. 

#### 3.1.4. Skin Elasticity

Skin elasticity represents the ability of the skin to resume its original shape once it is stretched [30]. This property is determined by elastic and collagen fibers, arranged as a mesh in the dermis, which are responsible for the elastic and mechanical properties of the skin, respectively. Specifically, collagen fibers determine the mechanical stability of the tissue and its resistance to deformation; meanwhile, elastic fibers restore deformed collagen bundles to a more relaxed state. As with all organs, skin health is affected by aging, but in two ways: chrono-aging (the physiological aging of the skin genetically determined) and photo-aging (due to ultraviolet (UV) exposure). These processes are also influenced by many other factors (e.g., smoke, alcohol, pollution), which lead to degradation of collagen and elastin with a more stiffness of the skin [31]. The resulting loss of elasticity, with consequent reduction in moisture and formation of wrinkles, typically occurs in sun-exposed areas, such as facial skin.

To evaluate the appropriateness of skin elasticity as an OV for the protection of skin against dehydration, database 3 was generated (see Table 1).

The measurement of the viscoelastic properties of the skin can be an indicator for the biological age of the skin. Being an elastic material, skin is subject to the mechanical laws defining its properties, which are modified by some factors. One of the most important is the process of skin aging (i.e., intrinsic and extrinsic). In fact, tensile functions of the skin and subcutaneous tissues contribute to the appearance of the aged and photo-damaged skin, and to the effects of various other pathophysiological processes [32]. Moreover, the depth of skin wrinkles can appear deeper in less moisturized skin.

Therefore, skin elasticity is not an appropriate outcome variable to be used alone for the substantiation of health claims in the context of protection of the skin against dehydration. However, it can be used in combination with more significant skin measures, such as TEWL, in order to provide significant and descriptive information about the hydration of the skin.

##### Cutometer^®^

To date, several methods based on different principles have been proposed for evaluating skin elasticity.

Among these, the Cutometer^®^ (Courage-Khazaka Electronic, Cologne, Germany) is probably the most commonly used instrument, and is considered the tool against which innovative or pilot instruments are to be compared. It is a handle device able to measure the viscoelastic properties of human skin using the suction/elongation method [33]. In detail, it can determine the elasticity of the upper skin layer using negative pressure, which is created in the device, and which mechanically deforms the skin. Skin is drawn into the aperture of the probe, and is released after a defined time. The depth of penetration of the skin inside the probe is determined using a non-contact optical measuring system.

The elasticity, i.e., the skin’s ability to return to its original position when deformed, is displayed as a strain-time curve that can be analyzed by software. The many parameters that can be measured include the ability to return to the original state, the overall elasticity, and the net elasticity of the skin [34]. In addition, F- and Q-parameters can be measured, giving additional indications on skin age and elasticity [35].

These parameters have been widely used in studies both on normal skin (including cosmetological applications) and in skin disease (e.g., psoriasis and scleroderma), reflecting both the state of the skin and the change in the skin structure. 

Because of significant regional variations in the viscoelastic properties of the skin, skin elasticity should be measured in the same area within a RCT [36].

In conclusion, the Cutometer is an appropriate method for the measurement of skin elasticity.

#### 3.1.5. Corneocyte Adhesion

The major purpose of epidermal differentiation is generating the *stratum corneum* (the superficial skin layer), whose primary function is to protect the internal organs from desiccation and from external injuries.

Moreover, *stratum corneum* functions as a protective barrier against the outside, because of its well-organized structure. The structural integrity of the *stratum corneum* is guaranteed by corneocytes—dead differentiated keratinocytes, in which the cytoplasm is constituted by specialized proteins (keratins, filaggrin)—while the interstices between the corneocytes are enriched by lipids [37].

*Stratum corneum* lipids are nearly devoid of phospholipids, and are selectively enriched in sphingolipids (ceramides), free sterols and not-esterified fatty acids, with lesser quantities of nonpolar lipids and cholesterol sulphate. Ceramides are hydrophobic, and are ideal for preventing excess water loss. Corneocyte adhesion is possible because they are bridged together through particularly modified desmosomes, named corneodesmosomes, which ensure the stability and integrity of the *stratum corneum*. As corneocytes migrate towards the upper layer of the *stratum corneum*, corneodesmosomes weaken their adhesive function, allowing cells to be disrupted, and promoting the physiological desquamation process for the renewal of the *stratum corneum* layer. Accelerated corneodesmosome degradation leads to an enhanced desquamation process, leaving the outer layer exposed to the environment, xerosis, desquamation, and possible penetration by microbes or allergens [38]. Abnormality of the normal corneodesmosome structure is also related to common skin disease (e.g., psoriasis, atopic dermatitis, lichen planus).

To evaluate the appropriateness of corneocyte adhesion as an OV for the protection of skin against dehydration, database 4 was generated (see Table 1).

Ultraviolet, the outermost epidermal layer, plays a critical role in the physical protection of the body. To maintain a constant SC thickness, as observed in normal epidermis, the continuous generation of corneocytes is balanced by cell shedding at the external surface in the tightly regulated process of desquamation. Cohesion of the SC is largely dependent on modified desmosomes or corneodesmosomes. Corneodesmosome degradation is of major importance in the desquamation process [39]. Several skin conditions are related to an altered skin scaling. In xerosis and various hyperkeratotic states, including psoriasis, accumulation of scales is observed, and the number of corneodesmosomes persisting over the corneocyte surface in the upper SC is greatly increased [40]. Moreover, some of the disorders of cornification, such as congenital ichthyosiform erythroderma, shows other alterations of the epidermal features, such as increased TEWL. Perturbed barrier function, abnormal desquamation, and hyperproliferation appear to be intimately linked.

In conclusion, corneocyte adhesion is not appropriate to be used alone for the substantiation of health claims in the context of protection of the skin against dehydration. Nevertheless, it can be used as supportive evidence when combined with other appropriate outcome measures (e.g., TEWL).

##### Squamometry

The assessment of scaling and dryness is difficult to standardize. Nowadays, several methods based on different principles have been proposed for the evaluation of skin scaling. The most common technique is based on the sampling of the superficial scaling portion of the *stratum corneum* using various kinds of adhesive tapes (for example D-Squame^®^ , CuDerm, Dallas, TX, USA) [41]. The quantification of scales or squames is made by using low-power imaging techniques [42], which allow the calculation of the squame size, number, optical density, and heterogeneity in relation to different aspects of the desquamation process. Image analysis can be performed using appropriate video-cameras such as the Visioscan^®^ (Courage-Khazaka Electronic, Cologne, Germany), an ultraviolet A (UVA)-light video camera with high resolution. The images show the skin structure and the level of dryness, but it can also be used on spots of hair and scalp. The camera can be connected to the computer, and several skin surface parameters can be determined, including desquamation, sebum production, scaliness, smoothness and roughness [43]. The technique is repeatable and reproducible.

In conclusion, squamometry is an appropriate method to use for the evaluation of corneocyte adhesion.

#### 3.1.6. Ceramide Concentration of the *Stratum Corneum*

In the *stratum corneum,* the matrix between corneocytes is composed of lipids arranged in numerous lamellar sheets, creating an impermeable barrier against external pathogens, and preventing water loss. More than 50% of the lipids of the intercellular space is represented by ceramides, which chemically belong to the sphingolipid class, as they are produced following the hydrolysis of sphingomyelin. Ceramides allow the formation of a barrier against cell permeability, and play a role in signal transduction, cell regulation, cell differentiation and immune response [44]. In human cells and tissues, there are commonly three ceramide classes, and human *stratum corneum* is known to contain even more complex ceramides. At least nine classes of ceramides have been classified on the basis of the characteristic fatty acid chain: non-hydroxy, α-hydroxy, ester-linked ω-hydroxy fatty acids, etc. [45] A lower level of ceramides, due to fatty acid hydrolysis, has been related to a loss in permeability of the skin surface, enhancing the skin inflammation by external agents. In fact, the loss of ceramides allows the pauperization of the intercellular lipid content, triggering water loss, as well as inflammation due to parasites and the grime accumulation between corneocytes. Although the causes of the decreased concentration of the ceramides in the *stratum corneum* due to skin lesions have been established, there is still a debate on the causes of the loss in non-lesioned skin, such as dermatitis, psoriasis or xerosis. However, it is widely recognized that skin disorders involving diminished barrier function show also a decrease in total ceramide content with some differences in the ceramide pattern.

To evaluate the appropriateness of ceramide concentration of the SC as an OV for the protection of skin against dehydration, database 4 was generated (see Table 1).

SC lipids are selectively enriched in sphingolipids (ceramides), free sterols, and free fatty acids, with lower quantities of non-polar lipids and cholesterol sulphate. Ceramide critical components of the barrier are hydrophobic, and are ideal for preventing excessive water loss. For this reason, SC water retention is improved by the addition of ceramides to damaged skin and a loss of these lipids causes profound barrier damage with a TEWL increase [44]. However, damage is also due to other mechanisms, such as a simultaneous, passive loss of extracellular calcium and potassium ions, and other lipids, including cholesterol and fatty acids, are also implicated. 

In conclusion, ceramide concentration of the SC cannot be used alone as an outcome variable for the substantiation of health claims in the context of protection of the skin against dehydration. However, it can be used as supporting evidence when appropriate outcome measures (e.g., TEWL) are also considered. In fact, all of these values are indirect values, so it is better to combine different measurements for a more reliable and sensitive result.

##### HPLC (High Performance Liquid Chromatography)

The most common method for detection and quantification of the different classes of ceramides is based on liquid chromatography techniques [46].

Biological samples that can be used for the analyses include epidermis collected by tape-stripping method, as well as blood or tissue samples (e.g., liver or adipose). Ceramides are chemically derivatized with benzoyl chloride or anhydride, depending on the fatty acid chain bound to sphingosine [47]. This step leads to the production of the *N*-acyl derivatives, which strongly absorb in the range 230–280 nm, depending on the ceramide type. Then, direct-phase HPLC is performed by using silica gels resins, while the mobile phase for the elution is based on organic solvents, such as hexane, pentane or ethyl acetate. The method is simple, sensitive and accurate. However, due to the instability of the derivatization product, samples should not be stored for prolonged periods, and analysis must be performed just after derivatization. Another limit of the HPLC method is that ceramide classes targeted for quantification are restricted to only few types.

In conclusion, HPLC is an appropriate method to be used for the measurement of the ceramide concentration of the *stratum corneum*.

#### 3.1.7. Pruritus

Pruritus, or itching, can be defined as an unpleasant sensation of the skin causing the desire to scratch the affected area. The mechanisms of pruritus have not yet been totally understood. It may be both localized and generalized and can occur as an acute or chronic condition. Itching can be the result of a dermatological disease (such as atopic dermatitis, eczema or urticaria), but can also be the result of several systemic diseases (e.g., thyroid or liver dysfunctions, important hormonal changes, hematological diseases, and nerve or psychiatric disorders) [48]. Nevertheless, itching can also be quite a common sign of simple skin conditions, such as dehydrated skin, and it can be exacerbated by cold and dry weather or increase with age. Moreover, if severe, itching can affect the quality of life of individuals and it has also important psychological implications [49,50]. Evaluating pruritus is very difficult due to the absolute subjectivity of the symptoms.

To evaluate the appropriateness of pruritus as an OV for the protection of skin against dehydration, database 6 was generated (see Table 1).

Pruritus is the most common symptom in dermatology. As mentioned above, pruritus can be related to several pathological conditions, but it can also indicate a lack of the normal skin integrity and correct hydration. For this reason, it is a frequent symptom in aged people [50]. Due to the large amount of pruritus-associated conditions, to the difficulties in identifying symptoms and the psychological components, and to the difficult quantification for its subjectivity, pruritus cannot be used alone as an outcome variable for the substantiation of health claims in the context of protection of the skin against dehydration. However, it can be used as supporting evidence when appropriate outcome measures (e.g., TEWL) are also considered.

##### Questionnaires

Itching is a subjective condition difficult to quantify. Moreover, its multifactorial etiology is difficult to characterize.

Several scales and scores have been proposed, both monodimensional and multidimensional. The former, including the visual analogue scale, numerical rating scale, and verbal rating scale, report information about intensity of itching in a specific moment. Conversely, multidimensional scales can provide different information including affected areas, progression of itch throughout the day and its evolution during the time, disability induced by the pruritus, improvement during treatment. This is why multidimensional scales can also be used as an instrument for evaluating treatment.

One of the most used methods are the visual analog scales (VAS), both vertical and horizontal [51]. Different authors have proposed different categorizations of VAS, such as: 0 = no pruritus, >0–<4 points = mild pruritus, ≥4–<7 points = moderate pruritus, 7–8.9 points = severe pruritus, and ≥9 points = very severe pruritus. The VAS is a valuable, easy and rapid method for estimating pruritus, and has shown good reproducibility and reliability [52]. In addition, VAS results have proven to be reproducible even in different populations and ethnic groups. However, similar to other monodimensional methodologies, VAS has some drawbacks. For example, it only measures intensity, without considering the impact on quality of life, or other parameters such as duration and distribution of itching. For this reason, a 5-D itch scale has been developed as a multidimensional questionnaire [53,54]. The five dimensions are degree, duration, direction, disability and distribution, with a scale specifically designed to facilitate the use of pruritus as an outcome variable in RCTs and clinical trials.

In conclusion, questionnaires are an appropriate method to be used for the measurement of pruritus. The use of VAS is appropriate, although the use of multidimensional methods is preferable, allowing a thorough comprehension of the pruritus condition.

#### 3.1.8. Water-Holding Capacity

The water in the skin is present as both free and bound (to macromolecules) water. 

The ability of the skin to hold water is primarily related to the SC, which acts as a barrier to water loss. Although the SC is a thin biological membrane, it is an essential part of the body, allowing it to survive even in a dry atmosphere by protecting our body from desiccation. The barrier function and the water-holding capacity of the SC depend on the presence of well-differentiated corneocytes with cornified envelope-associated proteins. These proteins bind the ceramide containing intercellular lipids and large amounts of moisturizing factors mainly consisting of water-soluble amino acids produced by enzymatic degradation of filaggrin [55].

Lipids and keratins play a key role in the formation of the permeability barrier, and are responsible for the water-holding capacity, which can be defined as the ability of the SC to retain water.

Because the SC is the interface between the fully hydrated viable epidermis and the dry atmosphere, there is a gradient of water in this thin biological membrane [56]. However, when the uppermost portion of the SC loses water, even normal individuals develop dry skin. This condition leads to a fine cracking in the SC, which deteriorates its barrier function focally, allowing permeation of external substances as well as TEWL.

To evaluate the appropriateness of water-holding capacity as an OV for the protection of skin against dehydration, database 6 was generated (see Table 1).

Skin hydration can depend on the water-holding capacities of the SC, but it is not a unique parameter for correctly assessing skin hydration. That is why the combination with other variables is important for a more accurate assessment of skin conditions and of potential alterations and changes. In conclusion, the water-holding capacity is not appropriate to be used alone for the substantiation of health claims in the context of protection of the skin against dehydration. However, it can be used as supporting evidence when other appropriate outcome measures (e.g., TEWL) are considered.

##### Corneometry

See “Corneometry” in Section 3.1.2.

#### 3.1.9. Skin Smoothness and Roughness

Due to the three-dimensional organization of cutis and subcutaneous tissue, skin surface is not perfectly smooth and is characterized by folds, furrows, orifices, and crests. In particular, some morphological characteristics of the epidermis (thickness of the cornified layer) and dermis (collagen content) can determine skin surface. However, pathological issues such as dermatitis, eczema, and other conditions, such as chronic light exposure, pollution and aggressive substances, may change the balance between skin smoothness and roughness, and may enhance the hardening process of the skin, leading to the coarsening of the skin surface structure with an increase of the wrinkle number and depth and, in general, of the roughness.

Skin smoothness and roughness, together with skin scaling and wrinkles, are considered to be the qualitative and quantitative parameters of skin physiological conditions, and represent the four clinical parameters that, if considered together, allow the Surface Evaluation of the Living Skin (SELS) [57].

To evaluate the appropriateness of skin smoothness and roughness as an OV for the protection of skin against dehydration, database 7 was generated (see Table 1).

With aging, skin texture undergoes several changes. Epidermal thickness decreases with a flattening of dermal-epidermal junction, while wrinkles increase due to loss of collagen content. This process is typical of aging, and it is more evident in photo-exposed areas. As the skin ages, changes in the texture and roughness or smoothness of the skin become apparent [58]. This is the result of loss of barrier integrity, which leads to increased water loss, but it is also due to changes in the collagen-supporting matrices showing visibly on the skin surface [59]. In fact, skin aging is a multifactorial process involving different mechanisms with a modification of different skin parameters, such as an increase in epidermal water loss.

In conclusion, skin smoothness and roughness are not appropriate to be used alone for the substantiation of health claims in the context of protection of the skin against dehydration. However, they can be used as supporting evidence when other appropriate outcome measures are considered (e.g., TEWL). In addition, the simultaneous evaluation of all 4 parameters, allowing the SELS (e.g., skin wrinkles and scaling), is preferable, in order to have a more reliable value.

##### UV Camera

Skin topography can be directly evaluated by using appropriate video-cameras. Among these, Visioscan^®^—a UVA-light video camera with high resolution—has been widely and successfully used as an alternative to the conventional color video cameras.

Visioscan^®^ consists of a black and white video sensor chip, an objective and an UVA-light, and is equipped with two special halogenide lights that illuminate the skin.

Visioscan^®^ can be used to measure the SELS, which has been proposed as a qualitative and quantitative measure of the skin surface. SELS includes evaluation of skin smoothness and roughness, skin scaling and wrinkles [57]. Other parameters can be analyzed with Visioscan^®^, such as skin desquamation and scalp and sebum production. With this method, skin can be optically monitored using an image-digitalization process. The grey level distribution is analyzed, making it possible to calculate the four SELS parameters, both quantitatively and qualitatively. This is useful to describe the skin surface as an index [60].

Visioscan^®^ is small, easy-to-handle, and economical, and makes it possible to have multi-frame pictures of the skin and to store these images for subsequent evaluation; it is therefore useful in elaborating different parameters throughout the time.

In conclusion, UV cameras such as Visioscan^®^ are an appropriate method for measuring skin smoothness and roughness, wrinkles and scaling. However, it is worth noting that none of these outcome measures is appropriate to be used alone for the substantiation of health claims in the context of maintenance of skin function. 

#### 3.1.10. Skin Wrinkles

Skin surface is not flat, but is characterized by several grooves, classified according to their depth. For this reason, the skin surface exhibits a network called microrelief or texture. This microrelief is very important for the mechanical properties of the skin, as it forms a complex system of small lines intersecting each other. On fingers and toes, these lines assume a characteristic disposition, and determine the fingerprint. As a result of age and UV exposure, skin microrelief changes, and some lines become more marked—being defined as wrinkles [58]. An example of wrinkles are so called “crow’s-feet”, which are localized on the forehead or around the eye circles, and may vary in amplitude and marking severity. 

Age, diseases (including psoriasis or atopic dermatitis) and the loss of hydration of the skin may alter the physiological desquamation of the skin, giving some roughness to the touch.

As already mentioned, skin wrinkles, together with skin scaling, roughness and smoothness, are considered to be qualitative and quantitative parameters of skin physiological conditions, and represent the four clinical parameters that, if considered together, allow the SELS [57].

To evaluate the appropriateness of skin wrinkles as an OV for the protection of skin against dehydration, database 8 was generated (see Table 1).

Wrinkle formation is a genetically-determined process (a process called “chrono-aging” or intrinsic aging). Several environmental factors contribute to wrinkle formation and/or worsening, such as smoking, UV-exposure, age, pollution (“photo-aging” or extrinsic aging) [58]. These mechanisms lead to a loss of elasticity that contributes to the worsening of wrinkles through decreased skin hydration. 

Even if skin wrinkles are a reliable parameter of skin structure, they are an indirect parameter of skin hydration [61]. For this reason, skin wrinkles are not appropriate to be used alone for the substantiation of health claims in the context of protection of the skin against dehydration. However, they can be used as supportive evidence when appropriate outcome measures are also considered (e.g., TEWL).

In addition, the simultaneous evaluation of all 4 parameters that allow the SELS (e.g., skin smoothness, roughness and scaling) is preferable, in order to obtain more accurate results.

##### UV Camera

See “UV Camera” In Section 3.1.9.

#### 3.1.11. Skin Scaling

Skin scaling, also known as skin peeling or desquamation, is the loss of the outer layer of the epidermis (*stratum corneum*) in large flakes. This layer consists of 18–20 layers of flattened dead keratinocytes with no nuclei and cell organelles, which have a defensive role against pathogens and environmental insults [62]. Skin scaling is a physiological process occurring when keratinocytes shed in an inappreciable way because of the new ones located in the underneath layer. In contrast, it is considered pathological when induced by disease or pathological causes, such as atopic dermatitis, contact dermatitis/eczema, or extreme cases of severe drug reactions, which can lead to a real exfoliation with loss of several layers of epidermis [63]. In these cases, keratinocytes scale down, leaving the outer layer of the skin unprotected. For these reasons, the pathological peeling of the epidermis may be accompanied by itch, dryness or irritating phenomena, which enhance the loss of the keratinocytes.

As already mentioned, skin scaling, together with skin wrinkles, roughness, and smoothness are considered to be qualitative and quantitative parameters of the skin physiological conditions, and represent the four clinical parameters that, if considered together, allow the SELS [57].

To evaluate the appropriateness of skin scaling as an OV for the protection of skin against dehydration, database 9 was generated (see Table 1).

Skin scaling is a sign frequently observed on the skin surface. It usually suggests an alteration in skin homeostasis and an alteration of the *stratum corneum*. During their transit through the epidermal layers toward the skin surface, keratinocytes follow a well predetermined differentiation program in order to originate the *stratum corneum*. Several conditions can lead to skin scaling. As cited above, some dermatological diseases are characterized by scaling. However, scaling can be also determined by a transient state of skin dehydration or other particular conditions. For example, being elderly is accompanied by an epidermal barrier impaired with TEWL increase, xerosis and skin scaling [64]. 

In conclusion, skin scaling is not appropriate to be used alone for the substantiation of health claims in the context of protection of the skin against dehydration. However, it can be used as supporting evidence when appropriate outcome measures are also considered (e.g., TEWL).

In addition, the simultaneous evaluation of all 4 parameters allowing the SELS (e.g., skin smoothness, roughness and wrinkles) is preferable.

##### UV Camera

See “UV Camera” in Section 3.1.9.

#### 3.1.12. Skin Tightness and Softness

Tightness and softness are skin conditions mainly dependent on skin compactness and elasticity, which may be influenced by several factors: diet; exposure to chemicals such as soaps or detergents; and atmospheric conditions, i.e., sun or wind. Pathological conditions, such as scleroderma, or diseases of collagen and subcutaneous tissue can modify skin tightness and softness [65].

To evaluate the appropriateness of skin tightness and softness as an OV for the protection of skin against dehydration, database 10 was generated (see Table 1).

Skin tightness and softness are features more typical of the deep tissue of the cutis (connective tissue) than the epidermis. They are determined by changes of deep components of the dermis, in particular, collagen and elastic fibers. Intrinsic and extrinsic aging leads to a degradation of collagen and elastic fibers with a consequent intensification of skin wrinkles, dryness and scaling.

However, skin texture can also be modified by pathological conditions of the connective tissue, which is characterized by changes in composition and/or tridimensional organization of collagen. In particular, systemic sclerosis (also known as scleroderma) or morphea (a localized form of sclerosis) are the result of an excessive activation of the repair process known as fibrosis leading to a severe skin tightening [66]. On the other hand, an increased softness is the main feature of elastic fiber disease, such as cutis laxa, in which the skin becomes inelastic and hangs loosely in folds. Patients develop a prematurely aged appearance. Nevertheless, a loss of skin elasticity is often associated with changes in other skin features, such as a decreased water content of the skin.

In conclusion, skin tightness and softness are not appropriate to be used alone for the substantiation of health claims in the context of protection of the skin against dehydration. However, they can be used as supporting evidence when appropriate outcome measures are also considered (e.g., TEWL).

##### Self-Assessment

Skin tightness or softness is evaluated by using the Modified Rodnan Score [67]. This score consists of an evaluation of skin thickness rated by clinical palpation using a 0–3 scale (0 = normal skin; 1 = mild thickness; 2 = moderate thickness; 3 = severe thickness with inability to pinch the skin into a fold) for each of the 17 surface anatomic areas of the body: face, anterior chest, abdomen, (right and left separately) fingers, forearms, upper arms, tights, lower legs, dorsum of hands and feet. These individual values are added, and the sum is defined as the total skin score, ranging from 0 to 51.

Modified Rodnan score is frequently utilized to evaluate the systemic sclerosis (or scleroderma), characterized by a progressive increase in skin tightness [68], and to monitor the efficacy of treatments. Skin thickness is used as a surrogate measure of disease activity, severity and mortality in patients with diffuse cutaneous systemic sclerosis, and higher skin thickness progression rates are predictive of internal organ involvement and mortality. This score can be used by physicians, but can also be self-assessed by the subjects, due to the high level of correlation between subject self-assessment and physician-assessment. In conclusion, the self-assessment with Modified Rodnan Score is an appropriate method to be used for the measurement of the skin tightness and softness.

#### 3.1.13. Skin Reddening and Erythema Formation

Erythema is a skin condition characterized by redness and is typically caused by vasodilation of superficial capillaries in the dermis [69]. It is a major feature of inflammatory skin reactions elicited by irritants or allergens. Erythema is often associated with an impaired barrier function, such as in the case of “sensitive skin” and rosacea, atopic dermatitis, psoriasis, allergic reactions, autoimmune disease [70]. Erythema is one of the most frequent signs of a pathological skin, with or without an impaired epidermal barrier function.

To evaluate the appropriateness of skin reddening and erythema formation as an OV for the protection of skin against dehydration, database 11 was generated (see Table 1).

If severe, reddening and erythema may lead to the loss of the barrier function of the skin. Moreover, an impaired epidermal barrier (as measured by an increase in TEWL) can be associated with clinical symptoms like redness and erythema.

Nevertheless, erythema is not directly and unequivocally related to an impaired structure or hydration or permeability function of the skin.

Therefore, skin reddening and erythema formation are not appropriate to be used for the substantiation of health claims in the context of protection of the skin against dehydration. 

#### 3.1.14. Capillary Blood Flow

The total blood flow within the systemic circulation is about 5 L/min. Most of the cardiac output is received by the gastrointestinal system and the skeletal muscle, while only ~5% goes to the skin. Cutaneous microcirculation is organized into two horizontal plexuses: one more superficial, situated in the upper dermis (1 ± 1.5 mm below the skin surface) and one deeper, at the dermal-subcutaneous junction [71]. Several factors can induce an alteration of cutaneous microcirculation with a vasodilation such as drugs or chemicals, UV, cutaneous inflammatory disease (e.g., psoriasis, eczema, allergic reactions, rosacea), and vascular disorders (i.e., Raynaud’s phenomenon or peripheral circulating disorders) [72]. A preserved microcirculation is essential for the proper delivery of oxygen and nutritive substances to the biological tissue as well as for the removal of toxins.

To evaluate the appropriateness of capillary blood flow as an OV for the protection of skin against dehydration, database 12 was generated (see Table 1).

A normal blood flow allows an appropriate delivery of nutrients and a simultaneous removal of products of metabolism. This is why an inappropriate removal of these waste products, which may occur in cases of reduced blood flow, could have negative effects on both the body and the skin.

Therefore, the measurement of blood flow might provide useful information on skin health. For instance, blood flow in the capillaries can be directly associated with wrinkle formation by a reduction of the flow and of the delivery of nutritional compounds to the cells [73].

However, there is no strong scientific evidence suggesting that a reduced capillary blood flow is unequivocally related to an impaired structure/hydration or elasticity of the skin. There are several other mechanisms that contribute to unhealthy skin. Erythema and altered blood flow are also not always associated with either an impaired of barrier or with a decrease in hydration. 

In conclusion, capillary blood flow is not an appropriate outcome variable to be used for the substantiation of health claims in the context of protection of the skin against dehydration. 

### 3.2. Protection of the Skin against Oxidative (Including UV-Induced) Damage

In physiological conditions, the skin cellular redox processes constantly produce free radicals, such as reactive oxygen species (ROS) and reactive nitrogen species (RNS), which are finely counteracted by endogenous and exogenous systems. When these antioxidant defenses are inadequate to fully inactivate the ROS (because of excessive production of ROS and/or because of inadequate antioxidant defenses), a condition of oxidative stress can occur. Oxidative stress has been widely associated with an increased risk of many acute and chronic diseases, mainly because of its role in altering the molecular structure and function of DNA, proteins and lipids. In addition, these oxidative products may accumulate over the time.

As skin is the most exposed organ to environmental sunlight and pathogens, it represents the major protective interface between the body and the environment. Therefore, it is exposed to many sources of oxidative stress including pollutants, infrared irradiation, xenobiotics and most of all UV radiation. Many mechanisms are involved in UV-induced damage, including up-regulation of gene expression through intracellular signal transduction pathways, suppression of immune reaction and induction of tolerance to antigens. Moreover, UV radiation seems to form a complex interaction with mitochondria, where it may contribute to a vicious circle of increasing damage.

#### 3.2.1. Oxidative Damage to DNA

Exposure to UVA and ultraviolet B (UVB) radiation may lead to the oxidation of DNA, proteins and lipids. As a result, several products of oxidation may originate. The main classes of products directly deriving from DNA oxidation are cis-syn cyclobutane pyrimidine dimers (CPDs), followed by pyrimidine-pyrimidone photoproducts [74].

Regarding the DNA products of oxidative DNA damage due to ROS, these mainly consist of a large group of compounds deriving from the DNA bases such as the guanine. One of the main products of DNA oxidation is the 8-hydroxydeoxyguanosine (8-OHdG), a pre-mutagenic lesion in mammalian cells that is considered a ubiquitous marker of oxidative stress. 8-OHdG is generated by hydroxyl radical, singlet oxygen, or direct electron transfer which does not involve any ROS. 8-OHdG may cause mutation (G:C to T:A) at DNA replication. In addition to 8-OHdG, UV radiation may produce many other products of DNA damage, such as protein-DNA crosslinks and single-strand breaks.

To evaluate the appropriateness of oxidative damage to DNA as an outcome variable for the protection of the skin against oxidative damage, database 13 was generated (see Table 1).

Solar UV radiation is one of the most important causes of skin lesions and related diseases. In fact, UV radiation induces a variety of photoproducts in DNA, including CPDs, pyrimidine-pyrimidone photoproducts, thymine glycols, cytosine damage, purine damage, DNA strand breaks, and DNA-protein crosslinks. If unrepaired damage occurs to regulatory genes (e.g., tumor suppressor genes), this may promote the process of carcinogenesis. In this context, gene mutation and activation may be important [75]. Other responses resulting from UV ray exposure of cells include increased cellular proliferation, which could have a tumor-promoting effect on genetically altered cells, as well as changes in components of the immune system present in the skin.

It is well documented that, in contrast to UVB radiation, the less energetic UVA photons may indirectly affect membranes, proteins, and DNA, by producing ROS. Among the most representative DNA oxidation products, 8-OHdG is commonly used as an indirect biomarker of oxidative DNA stress, although several factors (e.g., artifacts during isolation of DNA and its preparation for analysis) should be carefully considered, as they can affect its measurement [76].

To gain a better understanding of the mutagenic and carcinogenic features of UVA radiation, the identification of other products of oxidatively-generated DNA damage requires further investigations. These products include DNA–protein crosslinks and adducts, which may be issued from the covalent binding of reactive aldehydes arising from the decomposition of lipid hydroperoxides to nucleobases.

In conclusion, direct measures of oxidative damage to DNA, such as CPDs, are appropriate to be used for the substantiation of health claims in the context of protection of the skin against oxidative (including UV-induced) damage. Conversely, indirect products of DNA oxidation such as 8-OHdG are not appropriate for the substantiation of health claims in the context of protection against oxidative damage to DNA when measured alone, but they could be used as supporting evidence.

##### Skin Biopsy and High Performance Liquid Chromatography

The in vivo evaluation of direct markers of oxidative damage to DNA must be assessed directly on the target tissue, which should be correctly harvested, treated and stored, prior to being analyzed. Skin biopsy is the procedure used to sample the outer layer of the derma, and may be performed using different techniques, according to the type of analysis, the size and the location of the part which should be sampled. Shave biopsy, performed with a scalpel or a razor blade, allows the harvest of a tangential part of the skin across the target place. Punch biopsy, performed with a circular blade, allows the harvest of a round or ellipsoidal part of the epidermis, dermis and subcutaneous fat close to the target place of the skin. Excisional biopsy is an incisional biopsy where the harvested sample includes the target place with the lesion or the mutation [77]. A particular skin collection may be performed by using stripes that allow the sampling of the lonely dead keratinocytes on the outer layer, or by using cotton pads to collect sebum or skin lipids.

Skin samples collected by biopsy can be used for the evaluation of products of oxidative damage to DNA, such as CPDs, detected by using several methods, including high performance liquid chromatography with electrospray ionization-tandem mass spectrometry, which represents the reference method. In vitro UVR radiation of standard nucleosides is performed to obtain a calibration curve useful for quantifying the products of oxidation, such as cis-syn and trans-syn CPDs, cyclobutane thymine dimers, as well as oxidized nucleosides, i.e., 1,3 dimethyluracile [78].

In conclusion, skin biopsy followed by high performance liquid chromatography is an appropriate method for the measurement of direct markers of oxidative DNA damage like CPDs.

##### Skin Biopsy and Immunohistochemical Techniques

The in vivo evaluation of direct marker of oxidative damage to lipids must be assessed directly on the target tissue, and skin biopsy is the most accurate procedure, as described in “Skin Biopsy and High Performance Liquid Chromatography” in Section 3.2.1.

In alternative to chromatographic techniques, oxidative damage to DNA can be measured by semiquantitative immunohistochemical ones.

After the biopsy is assessed, skin samples are washed in saline solutions and fixed in opportune buffers (i.e., formalin, formaldehyde, paraformaldehyde) and included in paraffin. Samples are then sectioned and stained with non-human primary monoclonal or polyclonal antibodies anti-CPD by using a direct or a sandwich enzyme-linked immunosorbent assay (ELISA) technique. In this last case, a secondary antibody, which may be bound to a biotin-streptavidin complex, is required to increase the signal of CPD detection. Alternatively, primary cells harvested from the biopsy samples may be cultured and allowed to grow in vitro. In this way, it is possible to harvest cells from the culture medium, and process them for immunohistochemical analysis with primary or secondary antibodies anti-CPD [79]. It has been demonstrated that cells containing CPDs have a shorter survival and tend to die quicker than non-damaged cells.

Indirect products of oxidation, such as 8-OHdG, are also assayed with immunohistochemical techniques. As described previously, the included skin cells are processed for staining with anti-8-OHdG antibodies. Further washing allows the elimination of the remaining sample, and then a secondary anti-primary antibody is added to the well. An enzyme catalyzing the production of a colored substrate is bound to the same secondary antibody. The quantification of the analyte is carried out by measuring the intensity of the immunostaining, and by subtracting the value of the background at a cell-free area of the slice. Immunostaining is measured by using microscopes or cameras equipped with computer presenting image analysis software [80]. 

The method results are more sensitive to both CPD and 8-OHdG than the chromatographic ones, because even very small amounts of antigen are stained with the antibodies. Nevertheless, its results are less specific, particularly when polyclonal antibodies are used, as cross-link phenomena may occur between molecules having similar structure.

In conclusion, based on these considerations, skin biopsy followed by immunohistochemical detection of CPD and 8-OHdG is an appropriate method for measurement of markers of oxidative DNA damage at skin level.

#### 3.2.2. Oxidative Damage to Lipids

Lipids are fundamental components of the skin surface and are present as both sebaceous and epidermal lipids. Although the lipid profile is characterized by a large inter-individual variation, squalene, sebaleic acid, linoleic acid, and cholesterol are the most represented.

Skin lipids are susceptible to oxidation through three different mechanisms: free radical chain oxidation, enzymatic oxidation, and non-radical, non-enzymatic oxidation [81].

Skin photo-oxidation, which is a consequence of exposure to UVA and UVB, may result in the production of many oxidation products, including squalene monohydroperoxyde and hydroperoxycholesterol. In addition, lipid peroxidation products such as hydroxyeicosatetraenoic acids (HETEs) and isoprostanes have been found to increase in human skin following UV exposure.

Lipid peroxidation (LPO) products tend to accumulate in the cellular membranes proportionally with the cumulative oxidative stress of the skin. High concentrations of LPO products perturb the integrity of the membranes, and thus of the cells involved. Lipid peroxides can be further decomposed to many reactive aldehydic species, such as malondialdehyde, 4-hydroxynonenal, hexanal, as well as other saturated and unsaturated aldehydes and ketones [82].

To evaluate the appropriateness of oxidative damage to lipids as an outcome variable for the protection of the skin against oxidative damage, database 13 was generated (see Table 1).

UV radiation induces many indirect photo-chemical effects in the skin. In this context, LPO is one of the major pathways by which photo-oxidative stress disturbs cell signaling, and promotes photo-carcinogenesis and photo-aging. Studies have shown a significant linear relation between UVB exposure recorded by the dosimeters and colorimetry parameters of the skin reaction. 

An established marker of oxidative damage to cell membranes, which is reliably measured by immunohistochemistry, is represented by F2-isoprostanes. F2-isoprostanes are a series of prostaglandin F2-like compounds produced in vivo independently of cyclo-oxygenase as products of radical catalyzed LPO. Among these F2-isoprostanes, 8-epi-prostaglandin 2a (8-isoprostane) is the most representative. A linear relation was found between the generation of 8-isoprostane in the skin and the dosimeter readouts [83]. 8-Isoprostane has been validated as a marker for oxidative stress in various conditions and in the skin.

In conclusion, products of oxidative damage to lipids, such as isoprostanes, are appropriate outcome variables to be used for the substantiation of health claims in the context of protection of the skin against oxidative (including UV-induced) damage.

##### Skin Biopsy and Liquid or Gas Chromatography-Mass Technique

The in vivo evaluation of direct markers of oxidative damage to lipids must be assessed directly on the target tissue, and skin biopsy is the most accurate procedure, as described in “Skin Biopsy and High Performance Liquid Chromatography” in Section 3.2.1.

The most common method for detecting and quantifying hydroxyperoxides, such as squalene monohydroperoxide and cholesterol, is based on the liquid or gas chromatography technique [84]. After the collection of the skin, samples are extracted with organic solvents, e.g., acetone or 1-butanol, and prepared for the injection into chromatographic instruments. Chromatographic detection and quantification of target compounds may be performed by analyzing their mass, and/or that of their main fragments. Gas chromatography is more frequently used than liquid chromatography, as the mobile phase is cheaper, and because of the volatility of the sampling compounds.

Liquid chromatography techniques can be also used for the detection and quantification of isoprostanes (i.e., 8-Isoprostaglandin F2α), leukotrienes (i.e., HETE), hydroperoxyeicosatetraenoic acid (HpETEs) and lipoxin (i.e., lipoxin A4 (LXA4) and B4 (LXB4)) [85]. This is the preferred method, as it is simple, cheap, and very sensitive, as the concentration of these analytes in tissues are very scarce.

In conclusion, liquid or gas chromatography-mass techniques following skin biopsy are appropriate methods to be used for the measurement of markers of lipid oxidation like isoprostanes at skin level.

##### Skin Biopsy and Immunohistochemical Techniques

The in vivo evaluation of direct markers of oxidative damage to lipids must be assessed directly on the target tissue, and skin biopsy is the most accurate procedure, as described in “Skin Biopsy and High Performance Liquid Chromatography” in Section 3.2.1.

The detection and quantification of isoprostanes (i.e., 8-Isoprostaglandin F2α), leukotrienes (i.e., HETE, HpETEs and lipoxin (i.e., LXA4 and LXB4) can be performed by using immunohistochemical techniques, such as ELISA assays [83]. After the biopsy is assessed, skin samples are washed in saline solutions and fixed in opportune buffers and included in paraffin. Subsequently, samples are sectioned and stained with non-human primary antibodies specific for the marker (i.e., polyclonal goat anti-8-epiPGF-2α antibody). As described in “Skin Biopsy and Immunohistochemical Techniques”in Section 3.2.1, further washing allows the elimination of the remaining sample before a secondary anti-primary antibody is added in the well and an enzyme catalyzing the production of a colored substrates is bound to the same secondary antibody. The quantification of the analyte is carried out by measuring the intensity of immunostaining by subtracting the value of the background at a cell-free area of the slice. Immunostaining is measured by using microscopes or cameras equipped with a computer running image analysis software.

The principal limitation of immunohistochemistry is the need for high-resolution image analysis to obtain reliable results. Furthermore, the technique is more specific when monoclonal antibodies are used for the precise marker compared to polyclonal ones, which may recognize similar epitopes of different isoprostanes. However, monoclonal antibodies are expensive and request long times of production.

In conclusion, skin biopsy, followed by immunohistochemical detection of lipids, is an appropriate method for the measurement of markers of oxidative damage to lipid like isoprostanes in the skin.

#### 3.2.3. Oxidative Damage to Proteins

UV radiation can interact with cellular photosensitizers to generate ROS and ROS-mediated oxidative damage to DNA, proteins and lipids. It is well known that UVB rays induce a direct formation of DNA-photoproducts that are generally removed by the nucleotide excision repair system. UVA represents more than 95% of the incident solar radiation. The effects of UVA reflect the induction of oxidative stress that causes extensive protein oxidation.

Moreover, sustained UV-exposure can lead to a high extent of protein oxidation, which is generally increased in aged tissue [86]. ROS-induced oxidative damage to the structural dermal proteins collagen and elastin can result in changes in the protein conformation and unfolding, leading to modifications in the mechanical properties of skin. Collagen degradation and abnormal elastin accumulation are visible in photo-aged skin. Proteins undergo modifications and subsequent conformational changes when certain amino acids are converted to their oxidized forms.

Several biomarkers have been used to assess the extent of oxidative damage to protein in the skin. Reactive oxygen species (ROS) can lead to protein modifications occurring at the backbone, at amino acid side chains, as well as by the formation of protein carbonyls. Oxidative damage to proteins results in a multitude of products, arising from modification of a wide range of amino acids [87]. The various amino acid residues do not have the same susceptibility to oxidative modifications. For instance, histidine, leucine, methionine, and cysteine, as well as phenylalanine, tyrosine, and tryptophan, are more susceptible than others to the presence of thiols or hydroxyl moieties, which are more sensitive to oxidation processes [75].

Conversely, oxidation of proteins containing proline, arginine, lysine and threonine results in the formation of irreversible carbonyl groups. If compared to the deeper layers of the epidermis, these carbonyls have been found to be more concentrated in the SC.

To evaluate the appropriateness of oxidative damage to proteins as an outcome variable for the protection of the skin against oxidative damage, database 13 was generated (see Table 1).

Despite the presence of antioxidant defensive-systems, UV radiations cause extensive protein modification, which seems to be involved in aging processes and disease development [88]. 

ROS generated from UV exposure is the most important triggering agent of protein oxidation. Hydroxyl-, peroxyl-, nitro-, etc. radicals are able to modify both the carbon skeleton and the R group of the protein in order to create more unstable products. In detail, cysteine or methionine sulfenic/sulfonic acids, hydroxytyrosine, nitrotryptophan are produced inside the cell, causing the loss of the function of the relative proteins which they constitute. Due to their instability, the next step is the generation of protein carbonyl groups which are usually the resulting marker of protein oxidation, as they represent an irreversible form of protein modification [75]. 

In conclusion, products of oxidative damage to proteins such as oxidative changes in amino acids are appropriate outcome variables to be used for the substantiation of health claims in the context of protection of the skin against oxidative (including UV-induced) damage.

Conversely, protein carbonyls should be used in combination with direct markers of oxidative damage to proteins in vivo.

##### Skin Biopsy and Liquid Chromatography-Mass Technique

The in vivo evaluation of direct markers of oxidative damage to proteins must be assessed directly on the target tissue, and skin biopsy is the most accurate procedure, as described in “Skin Biopsy and High Performance Liquid Chromatography” in Section 3.2.1. 

A sensitive and specific method for detection of the direct products of protein oxidation is based on the liquid chromatography technique [89]. After the collection of the SC samples by using strips, they are washed and treated to avoid contaminations. Then, the sample is treated to avoid artefacts, and is digested with proteases, i.e., pepsin, trypsin and chymotrypsin, in acid conditions in order to avoid the formation of polymers. Samples are injected into chromatography instruments, and the detection and quantification of target compounds may be performed by analyzing the mass of the target compounds and/or their main fragments.

In conclusion, based on these considerations, skin biopsy followed by liquid or gas chromatography techniques is an appropriate method for the measurement of markers of oxidative damage to proteins at skin level.

### 3.3. Protection of the Skin from UV-Induced (Other Than Oxidative) Damage

#### 3.3.1. DNA Damage after UV Radiation Exposure

Exposure of skin to UV radiation has been shown to have a number of deleterious effects, including photo-aging, photo-immunosuppression and photo-induced DNA damage, which can lead to the development of skin cancer [90]. DNA has been shown to be a skin chromophore, and absorption of UV radiation by DNA can result in the formation of thymine dimers.

To evaluate the appropriateness of DNA damage after UV radiation exposure as OV for the protection of the skin from UV-induced damage, database 14 was generated (see Table 1).

DNA, in vitro and in vivo, is susceptible to being damaged when exposed to high-energy radiation. This damage can be a breaking of one or both of the strands in the DNA helix, a fusing of the two strands to each other, to themselves (dimers), or other types of molecular damage to the nucleotides. Usually, the majority of DNA damage is repaired. However, incomplete or deficient repair may lead to skin cancer, which is a multistep process involving tumor initiation, tumor promotion, and tumor progression, ultimately resulting in visible skin cancer [91]. Based on these considerations, the measurement of DNA damage after UV radiation exposure is an appropriate outcome variable to be used for the substantiation of health claims in the context of protection of skin from UV-induced (other than oxidative) damage.

##### Immunohistochemical Techniques

Methods for quantifying photoproducts in human skin include DNA extraction analysis and immunohistochemical analysis. In vitro fragmented DNA can be observed directly using conventional techniques such as capillary electrophoresis and the comet assay. The measurement of internal damage typically requires analysis such as high performance liquid chromatographic-mass spectrometry, hydrolysis of DNA followed by chromatographic separation, electrochemical measurements, or the enzymatic conversion of photoproducts into strand breaks [92]. However, in vivo quantification is more appropriate for evaluating UV-induced DNA damage in humans. There are some advantages to using immunohistochemical quantification, compared to DNA extraction. Histology is able to identify the skin compartment in which DNA damage has occurred, to which depth cells are affected, and which subpopulations of cells are damaged. Immunohistochemistry is based on the quantification of the thymine cyclobutane dimer (TT-CPD), the main DNA lesion induced by both UVB and UVA radiations. The level of TT-CPD in DNA may be determined by, immunohistochemical staining of photoproduct positive nuclei of keratinocytes in the epidermis. Manual counting of photoproduct-positive immunohistochemically stained nuclei in the epidermis is a frequently used method for quantification of DNA damage [93].

In conclusion, immunohistochemical analysis is an appropriate method for the measurement of DNA damage after UV radiation exposure.

#### 3.3.2. Depletion of Langerhans Cells after UV Light Exposure

Langerhans cells (LCs) are effective antigen-presenting cells that function as “custodians” of the skin, altering the immune system to pathogen entry, but also the tolerance to self-antigen and commensal microbes [94]. LCs are also recognized to play a key role in the induction and maintenance of the immune response against skin cancer. Exposure of human skin to solar UV light induces local and systemic immune suppression. It is known that alterations of numbers and immune functions of LCs mediate this phenomenon [95]. The effect of UV on epidermal LCs has been studied for many years, since the first reports in the 1980s focusing on the deleterious effects of UV on epidermal LCs in humans. Exposure to UV induces a decrease in LCs within the human skin, and this depletion is probably due to both cell death and enhanced migration to regional lymph nodes.

To evaluate the appropriateness of depletion of Langerhans cells after UV-light exposure as OV for the protection of the skin from UV-induced damage, database 15 was generated (see Table 1).

It has been shown that UV radiation induces epidermal LCs to emigrate to draining lymph nodes starting a few hours or a few days after UV exposure. This leads to a decrease in the number of Langerhans cells, which can persist for up to four weeks before recovery of a normal epidermal pool. The consequence is direct damage to the immunological function of the skin. Therefore, decreasing the depletion of Langerhans cells after UV light exposure is beneficial. It has been shown that the protection afforded by sunscreens against photo-immunosuppression must be broad-spectrum with an adequate UVA protection. Based on these considerations, the evaluation of depletion of Langerhans cells after UV light exposure, measured with appropriate techniques, is appropriate for the substantiation of health claims in the context of protection of skin from UV-induced (other than oxidative) damage.

##### Histochemical and Immunological Techniques

The measurement of the number of LCs necessarily requires skin samples. The simplest way to obtain a skin sample is a punch skin biopsy, which is generally performed in UV-unexposed skin such as buttock skin or the inner forearm after local anesthesia. This is an easy, minimally invasive and low-cost procedure, which provides the substratum for the subsequent measurement [77].

Langerhans cells cannot be identified in routinely prepared histologic testing, but they can be visualized at the light microscope by histochemical and immunological techniques. Appropriate methods for LC detection in the human skin include histo-enzymatic methods of adenosintriphosphatases, acid phosphatase, alpha-naphthylacetate esterase and the peroxidase-antiperoxidase immunochemistry method with S-100 protein antibody [96]. Based on these considerations, skin biopsy, if followed by a histochemical or immunological technique, is an appropriate method for the measurement of the number of LCs after UV-light exposure.

#### 3.3.3. UV-Induced Erythema and Erythema Grade (Reddening)

Erythema or skin reddening is an inflammatory response of the skin to UV-induced molecular and cellular damage [97]. If severe, i.e., sunburn, it may lead to blisters and loss of the several skin functions. Erythema and subsequent pigmentation are immediate responses of normal human skin exposure to UV radiation. Even if it is an indicator of direct UV-induced skin damage, there is no direct evidence of correlation with skin cancer and photo aging [98]. 

To evaluate the appropriateness of UV-induced erythema as OV for the protection of the skin from UV-induced damage, database 16 was generated (see Table 1).

A reduction in UV-induced erythema (e.g., measured as change in minimal erythema dose (MED) or erythema grade (reddening)) may indicate less UV-induced damage to the skin. However, it can also reflect a reduction in the capacity of the skin to react to molecular and cellular damage, so it does not represent a univocal measure of UV-induced damage at skin level. Moreover, erythema is a poor indicator of immunosuppression [99]. On the basis of these considerations, UV-induced erythema cannot be used alone as an outcome measure for the substantiation of health claims on protection of the skin from UV-induced damage. However, it can be used as supporting evidence when appropriate outcome variables are also used.

##### Minimal Erythemal Dose Test

The MED test allows the determination of the amount of UV radiation producing minimal erythema (sunburn or redness caused by engorgement of capillaries) of the skin within a few hours following exposure. The individual MED is determined by irradiating small areas of skin (usually sun un-exposed skin) with increasing UV doses. Results are read at 24 h post-exposure and the lowest dose in the series that only just produced erythema is considered to be the MED [100]. The increasing UV doses are determined in consideration of the specific skin phototype, since this strictly influences the intensity of the resulting erythema. This procedure is simple to perform, and requires only a few minutes. The only possible drawback is that an appropriate evaluation of phototype is fundamental to avoid excessive UV exposure time with consequent burning.

In conclusion, the MED test is an appropriate, simple and low-cost procedure for the measurement of UV-erythema.

#### 3.3.4. Delayed-Type Hypersensitivity Immune Response to Recall Antigens in the Skin

The delayed-type hypersensitivity (DTH) reaction is a cell-mediated reaction to several antigens injected in the skin. The DTH skin test is used to evaluate whether prior exposure to an antigen has occurred, and reflects the cell-mediated immunity that provides the main mechanism against fungi, viruses and other hosts. Following the injection of small amounts of antigen, a typical response occurs, including induration, swelling and monocytic infiltration into the site of the lesion within 24 to 72 h. This reaction has been considered a surrogate for immune response to tumor antigens [101]. Three types of DTH reactions have been described: contact hypersensitivity (e.g., caused by metal ions), tuberculin-type reaction and granulomatous hypersensitivity.

To evaluate the appropriateness of DTH immune response as an outcome variable for the protection of the skin from UV-induced damage, database 17 was generated (see Table 1).

Solar UV radiation has been demonstrated to have suppressive effects on the immune system. UV radiation inhibits antigen presentation and induces the release of immunosuppressive cytokines. This specific immunosuppression is mediated by antigen specific suppressor/regulatory T cells, which mediates UV-induced inhibition of DTH response in human skin.

However, DTH immune responses to recall antigens in the skin is more a marker of systemic UV-induced damage on the immune system rather than a marker of UV-damage at skin level. 

Therefore, based on these considerations, DTH immune responses to recall antigens in the skin cannot be used alone as an outcome measure for the substantiation of health claims on protection of the skin from UV-induced damage. However, it can be used as supporting evidence in combination with appropriate outcome variables.

##### Multitest Kit Merieux and Mantoux Testing

Most of the methods for determining the UV-induced immunosuppression are sunburn protection factor-based measures of UVB induced erythema response. The main drawback is the inability of these methods to provide an accurate evaluation of the immune protection. In this scenario, inhibition of DTH response has been suggested as a test for evaluating UV-induced immunosuppression, but the lack of appropriate techniques for evaluating immunosuppression still remains a challenge.

The DTH skin test is used to test if prior exposure to an antigen has occurred. So far, two procedures have been used successfully: Multitest Kit Merieux and Mantoux testing [102]. The Multitest Kit Merieux is a DTH to seven antigens (e.g., *Candida albicans*, *Streptococcus antigens*) and provides comprehensive information on the immune status of the human volunteers. Mantoux testing with tuberculin purified protein derivatives provides a possible alternative model of DTH to recall antigen.

Overall, the DTH response is not easy to assess; it requires several weeks, and it is invasive. However, it represents an interesting tool for evaluating photoimmunosuppression in subjects within RCTs.

In conclusion, Multitest Kit Merieux and Mantoux testing are appropriate methods for the measurement of DTH immune response to recall antigens in the skin.

## 4. Conclusions

Insufficient scientific substantiation for a health claim represents the most common reason for a negative response to a request for the authorization of a health claim. In this context, RCTs should be well-designed and well-performed, taking into account many parameters affecting the quality of a RCT, such as adequate sample size, proper study design (including an adequate duration of the intervention), adequate statistical analysis, and choice of appropriate OVs and related MMs. The present report provides a critical analysis of all the OVs and MMs that have been proposed so far in the context of maintenance of skin function, compliant with the European Regulation. This critical analysis could represent a useful tool for applicants during the design or selection of human intervention studies aimed to substantiate health claims related to skin function. Moreover, this information could serve as a basis for EFSA to develop and update the Guidance on the scientific requirements for health claims related to skin health.

## Figures and Tables

**Table 1:** Strategies used for retrieving the literature pertinent with outcome variables and methods of measurement related to maintenance of skin function.

DB Number	Syntax	Total Articles	Narrative Reviews	Systematic Reviews/Metanalyses	Validation Studies	Outcome Variables
1	“water loss, insensible” (mesh) OR “transepidermal water loss” (title/abstract) OR “twl” (title/abstract) OR “tewl” (title/abstract)) AND “english” (language) AND “humans” (mesh)”	2007	149	10	19	TEWL Water-holding capacity
2	“skin” (mesh) AND (“water” (mesh) OR “dehydration” (mesh) OR “dryness” (title/abstract) OR “hydration” (title/abstract)) AND “english” (language) AND “humans” (mesh)	1821	146	5	22	Skin hydration Skin dryness
3	“skin” (mesh) AND “elasticity” (mesh) AND “english” (language) AND “humans” (mesh)	585	35	3	18	Skin elasticity
4	(“corneocyte” (title/abstract) AND “adhesion” (title/abstract)) AND “english” (language) AND “humans” (mesh)	14	2	0	0	Corneocyte adhesion
5	(“Ceramide” (title/abstract) OR “*Stratum corneum*” (title/abstract)) AND “english” (language) AND “humans” (mesh)	260	31	0	2	Ceramide concentration of the *SC*
6	(“pruritus” (mesh) OR “itch” (title/abstract)) AND “english” (language) AND “humans” (mesh)	8746	1186	131	32	Pruritus
7	“skin” (mesh) AND (“smooth*” (title/abstract) OR “rough*” (title/abstract)) AND “english” (language) AND “humans” (mesh)	1144	77	4	0	Skin smoothness and roughness
8	“skin aging” (mesh) OR (“skin” (mesh) AND “wrinkles” (title/abstract)) AND “english” (language) AND “humans” (mesh)	5185	1277	83	40	Skin wrinkles
9	“skin” (mesh) AND (“scal*” (title/abstract) OR “desquamate*” (title/abstract) OR “flak*” (title/abstract) OR “peel*” (title/abstract)) AND “english” (language) AND “humans” (mesh)	93	13	1	0	Skin scaling
10	“skin” (mesh) AND (“tight*” (title/abstract) OR “soft*” (title/abstract)) AND “english” (language) AND “humans” (mesh)	1620	203	16	7	Skin tightness and softness
11	(“erythema” (mesh) OR (“skin” (mesh) AND “redness” (title/abstract) OR “reddening” (title/abstract))) AND “english” (language) AND “humans” (mesh)	15,702	1479	93	29	Skin reddening and erythema formation
12	“capillaries” (mesh) AND “english” (language) AND “humans” (mesh)	11,390	1110	26	40	Capillary blood flow
13	“skin” (mesh) AND (“oxidative stress”(mesh) OR “oxidative damage” (title/abstract)) AND “english” (language) AND “humans” (mesh)	818	126	4	1	Oxidative damage to DNA Oxidative damage to lipids Oxidative damage to proteins
14	“skin” (mesh) AND “dna damage” (mesh) AND “english” (language) AND “humans” (mesh)	739	123	3	1	DNA damage after UV exposure
15	“langerhans cells” (mesh) AND “english” (language) AND “humans” (mesh)	3338	500	3	2	Depletion of Langherans cells after UV light exposure
16	“erythema” (mesh) OR (“skin” (mesh) AND “redness” (title/abstract) OR “reddening” (title/abstract)) AND “english” (language) AND “humans” (mesh)	15,702	1479	93	29	UV-induced erythema and erythema grade
17	“hypersensitivity, delayed” (mesh) AND “english” (language) AND “humans” (mesh)	15,141	2001	73	17	DTH immune response to recall antigens in the skin

Legend: DB: Database; DTH: delayed type hypersensitivity; SC: *stratum corneum*; TEWL: Transepidermal water loss; UV: ultraviolet.

## Abbreviations

CPDs	cyclobutane pyrimidine dimers
DTH	delayed type hypersensitivity
EFSA	european food safety authority
ELISA	enzyme-linked immunosorbent assay
HETE	hydroxyeicosatetraenoic acids
HpETEs	hydroperoxyeicosatetraenoic acid
HPLC	high performance liquid chromatography
LCs	langerhans cells
LPO	lipid peroxidation
LXA4	lipoxin a4
LXB4	lipoxin b4
MED	minimal erythemal dose
MM	method of measurement
OV	outcome variable
RCT	randomized controlled trial
ROS	reactive oxygen species
SC	stratum corneum
SELS	surface evaluation of the living skin
TEWL	transepidermal water loss
TT-CPD	thymine cyclobutane dimer
UV	ultraviolet
UVA	ultraviolet A
UVB	ultraviolet B
VAS	visual analog scales
VP	vapor pressure
8-OHdG	8-hydroxydeoxyguanosine
